# Supplementary material for: A fault-intrusion-tolerant system and deadline-aware algorithm for scheduling scientific workflow in the cloud
Source: PeerJ Comput Sci. 2021 Nov 2;7:e747. doi: 10.7717/peerj-cs.747 (PMC8576554; doi:10.7717/peerj-cs.747)
Supplement: Supplemental Information 6 [file peerj-cs-07-747-s006.zip › Myproject1.1/docs/org/cloudbus/cloudsim/Pe.html]

Pe


JavaScript is disabled on your browser.


- Overview
- Package
- Class
- Use
- Tree
- Deprecated
- Index
- Help

- Prev Class
- Next Class

- Frames
- No Frames

- All Classes

- Summary:
- Nested |
- Field |
- Constr |
- Method

- Detail:
- Field |
- Constr |
- Method


org.cloudbus.cloudsim

## Class Pe

- java.lang.Object
- - org.cloudbus.cloudsim.Pe

- ---

    

  ```
  public class Pe
  extends java.lang.Object
  ```

  CloudSim Pe (Processing Element) class represents CPU unit, defined in terms of Millions
  Instructions Per Second (MIPS) rating.  
  **ASSUMPTION: **All PEs under the same Machine have the same MIPS rating.****

  Since:
  :   CloudSim Toolkit 1.0

- - ### Field Summary

    Fields

    | Modifier and Type | Field and Description |
    | --- | --- |
    | `static int` | `BUSY` Denotes Pe is allocated and hence busy in processing Cloudlet. |
    | `static int` | `FAILED` Denotes Pe is failed and hence it can't process any Cloudlet at this moment. |
    | `static int` | `FREE` Denotes Pe is FREE for allocation. |
  - ### Constructor Summary

    Constructors

    Constructor and Description

    `Pe(int id,
    PeProvisioner peProvisioner)`

    Allocates a new Pe object.
  - ### Method Summary

    Methods

    | Modifier and Type | Method and Description |
    | --- | --- |
    | `int` | `getId()` Gets the id. |
    | `int` | `getMips()` Gets the MIPS Rating of this Pe. |
    | `PeProvisioner` | `getPeProvisioner()` Gets the Pe provisioner. |
    | `int` | `getStatus()` Gets the status of this Pe. |
    | `protected void` | `setId(int id)` Sets the id. |
    | `void` | `setMips(double d)` Sets the MIPS Rating of this Pe. |
    | `protected void` | `setPeProvisioner(PeProvisioner peProvisioner)` Sets the pe provisioner. |
    | `void` | `setStatus(int status)` Sets Pe status to either Pe.FREE or Pe.BUSY |
    | `void` | `setStatusBusy()` Sets Pe status to busy, meaning it is already executing Cloudlets. |
    | `void` | `setStatusFailed()` Sets this Pe to FAILED. |
    | `void` | `setStatusFree()` Sets Pe status to free, meaning it is available for processing. |

    - ### Methods inherited from class java.lang.Object

      `clone, equals, finalize, getClass, hashCode, notify, notifyAll, toString, wait, wait, wait`

- - ### Field Detail


    - #### FREE

      ```
      public static final int FREE
      ```

      Denotes Pe is FREE for allocation.

      See Also:
      :   Constant Field Values


    - #### BUSY

      ```
      public static final int BUSY
      ```

      Denotes Pe is allocated and hence busy in processing Cloudlet.

      See Also:
      :   Constant Field Values


    - #### FAILED

      ```
      public static final int FAILED
      ```

      Denotes Pe is failed and hence it can't process any Cloudlet at this moment. This Pe is
      failed because it belongs to a machine which is also failed.

      See Also:
      :   Constant Field Values
  - ### Constructor Detail


    - #### Pe

      ```
      public Pe(int id,
        PeProvisioner peProvisioner)
      ```

      Allocates a new Pe object.

      Parameters:
      :   `id` - the Pe ID
      :   `peProvisioner` - the pe provisioner
  - ### Method Detail


    - #### setId

      ```
      protected void setId(int id)
      ```

      Sets the id.

      Parameters:
      :   `id` - the new id


    - #### getId

      ```
      public int getId()
      ```

      Gets the id.

      Returns:
      :   the id


    - #### setMips

      ```
      public void setMips(double d)
      ```

      Sets the MIPS Rating of this Pe.

      Parameters:
      :   `d` - the mips


    - #### getMips

      ```
      public int getMips()
      ```

      Gets the MIPS Rating of this Pe.

      Returns:
      :   the MIPS Rating


    - #### getStatus

      ```
      public int getStatus()
      ```

      Gets the status of this Pe.

      Returns:
      :   the status of this Pe


    - #### setStatusFree

      ```
      public void setStatusFree()
      ```

      Sets Pe status to free, meaning it is available for processing. This should be used by SPACE
      shared hostList only.


    - #### setStatusBusy

      ```
      public void setStatusBusy()
      ```

      Sets Pe status to busy, meaning it is already executing Cloudlets. This should be used by
      SPACE shared hostList only.


    - #### setStatusFailed

      ```
      public void setStatusFailed()
      ```

      Sets this Pe to FAILED.


    - #### setStatus

      ```
      public void setStatus(int status)
      ```

      Sets Pe status to either Pe.FREE or Pe.BUSY

      Parameters:
      :   `status` - Pe status, true if it is FREE, false if BUSY.


    - #### setPeProvisioner

      ```
      protected void setPeProvisioner(PeProvisioner peProvisioner)
      ```

      Sets the pe provisioner.

      Parameters:
      :   `peProvisioner` - the new pe provisioner


    - #### getPeProvisioner

      ```
      public PeProvisioner getPeProvisioner()
      ```

      Gets the Pe provisioner.

      Returns:
      :   the Pe provisioner


- Overview
- Package
- Class
- Use
- Tree
- Deprecated
- Index
- Help

- Prev Class
- Next Class

- Frames
- No Frames

- All Classes

- Summary:
- Nested |
- Field |
- Constr |
- Method

- Detail:
- Field |
- Constr |
- Method
